# Role of Glucose-Lowering Medications in Erectile Dysfunction

**DOI:** 10.3390/jcm10112501

**Published:** 2021-06-05

**Authors:** Angelo Cignarelli, Valentina Annamaria Genchi, Rossella D’Oria, Fiorella Giordano, Irene Caruso, Sebastio Perrini, Annalisa Natalicchio, Luigi Laviola, Francesco Giorgino

**Affiliations:** Department of Emergency and Organ Transplantation, Section of Internal Medicine, Endocrinology, Andrology and Metabolic Diseases, University of Bari Aldo Moro, 70124 Bari, Italy; angelo.cignarelli@gmail.com (A.C.); valentina.genchi@uniba.it (V.A.G.); rossella.doria@uniba.it (R.D.); fiorella.giordano01@gmail.com (F.G.); ireneca91@gmail.com (I.C.); sebastio.perrini@uniba.it (S.P.); annalisa.natalicchio@uniba.it (A.N.); luigi.laviola@uniba.it (L.L.)

**Keywords:** erectile dysfunction, diabetes, glucose-lowering medications

## Abstract

Erectile dysfunction (ED) is a long-term complication of type 2 diabetes (T2D) widely known to affect the quality of life. Several aspects of altered metabolism in individuals with T2D may help to compromise the penile vasculature structure and functions, thus exacerbating the imbalance between smooth muscle contractility and relaxation. Among these, advanced glycation end-products and reactive oxygen species derived from a hyperglycaemic state are known to accelerate endothelial dysfunction by lowering nitric oxide bioavailability, the essential stimulus of relaxation. Although several studies have explained the pathogenetic mechanisms involved in the generation of erectile failure, few studies to date have described the efficacy of glucose-lowering medications in the restoration of normal sexual activity. Herein, we will present current knowledge about the main starters of the pathophysiology of diabetic ED and explore the role of different anti-diabetes therapies in the potential remission of ED, highlighting specific pathways whose activation or inhibition could be fundamental for sexual care in a diabetes setting.

## 1. Introduction

Erectile dysfunction (ED) is a common underestimated complication of diabetes mellitus that affects more than 50% of people with diabetes [1,2]. Diabetes dramatically raises the risk of developing ED by 2.5-fold. Additionally, ED represents an early sentinel of a cardiovascular event preceding a coronary event by at least three years [3], and thus warrants remarkable consideration by clinicians so as to prevent one of the main causes of death among subjects with type 2 diabetes (T2D) [4].

Several mechanisms related to the onset of diabetes may explain the high prevalence of ED among subjects with T2D. Hyperglycaemia and insulin resistance promote several biochemical derangements in the vascular and neurological systems, leading to an improper induction of erection. Moreover, obesity and visceral fat accumulation, typically observed in subjects with T2D, collectively represent one of the main risk factors for secondary hypogonadism [5]. Indeed, almost 40% of subjects with T2D are obese [6], and roughly 50% of these individuals have a haemoglobin A1c (HbA1c) concentration higher than 7% [7]. Therefore, the association of visceral obesity with the hyperglycaemic/dyslipidaemic milieu and the combination of low circulating testosterone levels and the development endothelial dysfunction, macrovascular and microvascular disease, and diabetic neuropathy can significantly alter the fine mechanisms involved in regular erectile function.

Several hypoglycaemic agents are commonly prescribed to subjects with T2D to reduce HbA1c levels and, thus, the incidence and progression of diabetic complications. Therefore, a significant impact of these drugs on erectile function is to be expected. However, substantial differences in the mechanism of action, glucose-lowering efficacy and effects on body weight and other cardiovascular risk factors of each drug may explain a potential variable impact on erectile function.

This review sought to report on the selective effects demonstrated by glucose-lowering medications on erectile function and explore the potential mechanisms involved.

## 2. Pathophysiology of ED

Erection results from nerve impulses that produce vascular and cavernosal smooth muscle relaxation, which lead to increased arterial inflow to the penile corpora cavernosa. This is predominantly mediated by nitric oxide (NO), which is mainly produced by parasympathetic noradrenergic and noncholinergic neurons and cholinergic neurons, which, in turn, stimulate endothelial cells. After the release of NO, vascular smooth muscle responds with a cyclic guanosine monophosphate (cGMP)-mediated dilatation of the corpora cavernosa, facilitating the supply of blood. Increasing the blood flow impedes venous return through passive compression of the subtunical venules, maintaining the erection. However, a lack of NO due to endothelial dysfunction triggers insufficient relaxation of the vascular smooth muscle of the corpora cavernosa, resulting in ED [8]. In this setting, several biological contributors are known to interfere with sexual performance, particularly in the context of metabolic illness (e.g., diabetes, obesity). We henceforth discuss current advances concerning the effects of endogenous mediators and related molecular mechanisms that affect erectile functionality and suggest potential therapeutic strategies that may restore metabolic control along with improved sexual function.

### 2.1. Hyperglycaemia

A higher glucose level is a hallmark of T2D and contributes to various metabolic derangements that promote endothelial dysfunction and vascular complications. Long-term hyperglycaemia reportedly leads to the increased generation of advanced glycation end-products (AGEs) and reactive oxygen species (ROS) [9,10,11,12], which may accelerate endothelial dysfunction through the impairment of endothelial NO synthase (eNOS) activity and NO production. As a result, an imbalance between vasoactive mediators and vasoconstrictors occurs, modifying the vascular permeability and resulting in a disruption of endothelial integrity. In this context, endothelial walls lose their sensitivity to mediators of vasodilation and increase their responses to vasoconstrictor stimuli, leading to in impaired vascular and smooth muscle relaxation, generating clinical ED [13].

**Figure 1:**
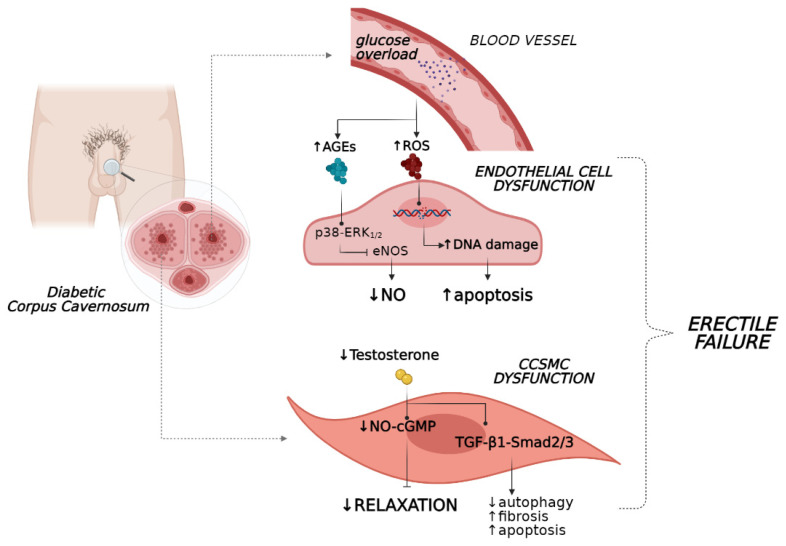
Effects of hyperglycaemia and hypogonadism on endothelial cell and CCSMC dysfunction. AGE, advanced glycation end-product; CCSMCs, corpus cavernosum smooth muscle cells; cGMP, cyclic GMP; eNOS, endothelial nitric oxide synthase; NO, nitric oxide.

The formation of AGE products represents a real insult that hampers endothelium functionality due to the abrogation of signalling pathways regulating NO release [14,15]. Indeed, when the production of AGEs was abrogated in vivo with tight glycaemic control, mice with type 1 diabetes (T1D) showed restored erectile parameters in association with an amelioration of histological features of the corpora cavernosum [16]. Additionally, in a recent study on the human coronary artery, endothelial cells stimulation with AGEs induced reductions in mRNA and protein levels of NOS, phospho-eNOS (Ser1177), and eNOS enzyme activity via activation of the p38–ERK1/2 pathway (Figure 1) [17]. These in vitro results confirmed previous findings where AGE levels were found to positively correlate with endothelial dysfunction in diabetes [18], typically in penile tissue [19].

Additionally, the maintenance of endothelial integrity needs the balance between the angiopoietin growth factor-1 (Ang-1), which favours vessel integrity and endothelial cell renewal through Tie-2 receptor activation [20], and the angiopoietin growth factor-2 (Ang-2), having a proangiogenic and proinflammatory action by inhibiting the same receptor [21]. In this scenario, both hyperglycaemia and AGEs are known to exert a detrimental effect on endothelial function by impairing the angiopoitin-Tie-2 system. A previous in vitro study revealed that high glucose concentrations suppress the ability of Ang-1 to activate the Tie-2 receptor with the abrogation of PI3K/Akt signalling, thus reducing vascular protection [22]. This event was found to be associated with a strong downregulation of the Tie-2 receptor, as shown in a mouse model of T2D [23]. Furthermore, hyperglycaemia together with AGEs impaired the intracellular signalling cascade induced by Ang-1 in a FoxO1-dependent manner, with a subsequent increase in Ang-2 production, thus enhancing the responsiveness of endothelial cells to inflammatory or angiogenic cytokines [24]. The enhanced production of Ang-2 was also observed in patients with T2D [25], whose circulating levels were found to correlate with the presence of vascular complications [26]. Therefore, a vicious cycle occurs where the impairment of Ang-1 signalling leads to an increased secretion of Ang-2 that compromises the Ang-1-Tie-2 axis thus destabilizing the vasculature integrity. Considering the critical role of the Ang-1/Ang-2 system in the preservation of endothelial function, it is reasonable that a glucose-induced failure of this axis could also affect the vascular bed of penile tissue thus favouring ED development.

The poor endothelium-dependent vasorelaxation in ED is also fostered by ROS. These factors show a deleterious effect on cavernosal smooth muscle reactivity, interfering with eNOS bioavailability. Indeed, several studies have demonstrated that the restoration of the anti-oxidant system via either pharmacological compounds or genetic methods re-established erectile function through an increase in NO production [27,28] and appeared to potentiate the efficacy of current pharmacological medications for sexual dysfunction [29].

Another source of oxidative stress is hydrogen peroxide (H_2_O_2_), whose levels were found to be enhanced in the penile tissue of animals prone to diabetic-induced ED. Particularly, H_2_O_2_ levels increases according to the progression of metabolic disease and generate reactive radicals, enhancing the risk of ED development [30]. Together, these processes caused direct injury to the endothelial system of the penis vasculature due to the accumulation of peroxide lipids species, DNA and protein modifications (i.e., nitration of tyrosine) [13], leading to aggravation of endothelial dysfunction and interference with erectile vasorelaxation (Figure 1). Additionally, when penile tissue malfunction persists, increased apoptosis of endothelial cells occurs together with a reduction in their regeneration, weakening the homeostasis of the vascular bed [31]. In this scenario, the persistence of hyperglycaemia also appears to have a critical role in erectile performance. This phenomenon appears to be associated with a ‘metabolic memory’ as was recently observed in men with T2D and ED, in which early exposure to sustained hyperglycaemia has long-term disadvantageous effects on erectile function, which persist even after patients achieved better glycaemic control [32].

Together, these results suggest that not only glucose overload but also the duration of exposure to a hyperglycaemic state under diabetic conditions could favour ED development to different extents.

### 2.2. Hypogonadism

Hypogonadotropic hypogonadism is common in men with T2D, with a prevalence of up to 40%. The major molecular player in the impaired hypothalamic–pituitary–testicular (HPT) axis is an increase in body fat mass. Longitudinal results from the European Male Ageing Study reported that obesity has a detrimental effect on testosterone release and indicated that modifications to the HPT axis were reversible following weight reduction [33].

In a multicentre population-based study, significant relationships between total testosterone level and worse sexual functioning and between free testosterone level and ED in ageing men were found [34]. Indeed, testosterone regulates sexual function and biological processes underlying erection via both central and peripheral mechanisms [35]. Therefore, the deprivation of circulating levels of this hormone compromises sexual drive and function, leading to ED development.

Compelling in vivo evidence suggests that androgens regulate corporeal haemodynamics and participate in the maintenance of penile tissue integrity. As a matter of fact, androgens tightly control endothelial cell proliferation and survival [36] and the penis vasculature via both direct and indirect mechanisms in humans [37,38]. Particularly, testosterone evokes vasorelaxation by stimulating NOS production and endothelium-dependent hyperpolarisation factors (EDHFs) [39]. Therefore, this hormone is essential for “erectogenesis” since its deprivation leads to enhanced endothelial cell apoptosis and decreased production of eNOS [40] and NO levels, as was recently observed in both human and rat studies (Figure 1) [41,42]. 

The vasoprotective effectiveness of androgens was also confirmed after testosterone replacement therapy (TRT), where endothelial function was completely restored and the progression of ED was significantly counteracted both for a short time [43] and in the long-term in men with hypogonadism [44]. Accordingly, men affected by hypogonadism with veno-occlusive dysfunction demonstrated improved penile haemodynamics after TRT with decreased end-diastolic velocity of the cavernous artery, thus preventing impotency [45]. Notably, the molecular processes driving ED amelioration after testosterone replacement were further investigated in vivo. Indeed, T1D rats with an experience of ED reverted hypogonadism after exposure to testosterone supplementation in association with restored expression of neuronal NOS (nNOS) and phosphodiesterase type 5 (PDE5) and ameliorated penile sensitivity to vasorelaxation stimuli [46].

Hence, adequate bioavailability of testosterone in erectile tissue is necessary to regulate the expression and activity of PDE5, the key target of current pharmacotherapy for ED [47,48]. Particularly, PDE5 catalyses the conversion of cGMP into GMP, thus inducing the contractility of smooth muscle cells (SMCs) and penile flaccidity. Therefore, the inhibition of PDE5 by selective blockers (PDE5i) can ameliorate ED through an increase in the intracellular cGMP concentrations, leading to vascular SMCs relaxation. The activity of these compounds, however, was completely lost in the presence of severe androgen deprivation. Indeed, under low testosterone levels, a typical condition of hypogonadal patients, reduced expression of PDE5 within penile tissue was observed, which, in turn, resulted in a failure of PDE5i-based therapy. When testosterone levels were normalised with TRT, PDE5 expression was restored, thus ameliorating PDE5i efficacy with final recovery of penile functionality and erectile performance [47,49]. Whether the effect of this androgen on PDE5 expression is direct or mediated by influence on other factors is still a matter of debate. Indeed, in a recent review, conflicting results emerged on the presence of a putative androgen response element (ARE) within the human and rat PDE5A gene sequence; furthermore, other studies showed that reduced PDE5 expression observed in castrated animals is due to a reduction of smooth muscle content rather than by direct effects of reduced androgen levels [50].

Despite the aforementioned results that described direct modulation of vasodilator genes by testosterone, emerging ex vivo data suggest that androgens regulate the physiological function of SMCs in human corpora cavernosum regardless of NO-cGMP dilator pathways. In particular, supraphysiological levels of testosterone evoked relaxation responses of SMCs in explants of human corpora cavernosum from patients with ED probably via ‘nongenomic mechanisms’ [51,52]. In particular, the treatment of precontracted cavernosal tissue with testosterone led to cell relaxation within a few minutes, even though both NO synthesis and androgen receptors were inhibited, suggesting the involvement of noncanonical mechanisms [51,52]. 

Noteworthy, inadequate testosterone bioavailability, beyond the detrimental effects of endothelial integrity, impaired normal erectile organ structure by favouring the loss of corpus cavernosum SMCs (CCSMCs) and increasing collagen deposition in the penis [53]. In this setting, the TGF-β1–Smad2/3 signalling pathway appears to mediate fibrotic processes of penile tissue in association with reduced autophagy and increased apoptosis of CCSMCs, as shown in a castrated rat model (Figure 1) [54]. These events were reverted by testosterone administration, which preserved regular erectile function and facilitated histology stabilisation of the penile organ [54]. In concert, these findings suggest that testosterone deprivation under eugonadal conditions contribute to the impairment of penile tissue and function.

## 3. Glucose-Lowering Medications 

The efficacy of current glucose-lowering medications on erectile functionality has not been extensively investigated. In particular, improvements in ED after anti-diabetes drug therapy may be ascribed to indirect mechanisms such as the reduction of hyperglycaemia, excess body weight, high blood pressure and the amelioration of other detrimental factors. Notwithstanding, a direct effect of glucose-lowering agents on both endothelial and smooth muscle cells is reasonable.

The pharmacotherapy of diabetes was recently enriched due to the availability of novel agents [i.e., glucagon-like peptide-1 receptor agonists (GLP-1RAs), sodium–glucose cotransporter-2 inhibitors (SGLT2is), dipeptidyl peptidase-4 inhibitors (DPP4is)] able to correct hyperglycaemia with less hypoglycaemic events. The effects of these molecules on erectile function, however, remain poorly investigated. Several studies have claimed that the protective effects on the vascular wall of these drugs are exerted via indirect mechanisms such as the reduction of glycaemia, blood pressure and body weight. Whether these anti-hyperglycaemic agents preserve penile endothelial cell viability and function, thus preventing diabetic-related erectile failure, however, is not known. Herein, we discuss the current knowledge on the effects of anti-diabetes agents on the restoration of sexual performance. 

### 3.1. Insulin

**Figure 2:**
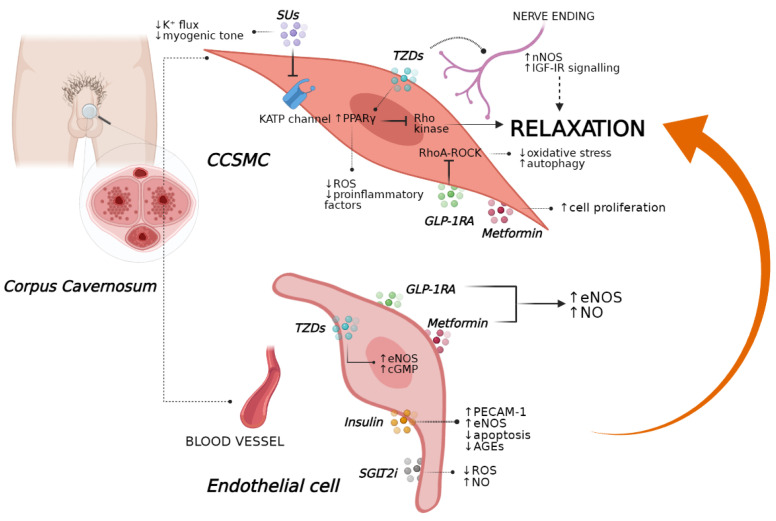
Principal glucose-lowering agents and their downstream pathways that could restore the relaxation of CCSMC and promote erectile function in a diabetic setting. AGE, advanced glycation end-product; AMPD, AMP deaminase; KATP; ATP-sensitive potassium channel; CCSMC, corpus cavernosum smooth muscle cell; cGMP, cyclic GMP; eNOS, endothelial nitric oxide synthase; GLP-1RA, GLP-1 receptor agonist; I4, SU compound; IGF-IR, insulin-like growth factor I receptor; MYPT1, myosin phosphatase target subunit 1; nNOS, neuronal nitric oxide synthase; NO, nitric oxide; PECAM-1, platelet endothelial cell adhesion molecule; ROS, reactive oxygen species, SGLT2i, sodium–glucose cotransporter-2 inhibitor; SUs, sulfonylureas; TZDs, thiazolidinediones. ↓ decrease, ↑ increase.

Insulin regulates several aspects of energy metabolism, including the absorption of nutrients in metabolically active organs (i.e., liver, skeletal muscle, adipose tissue) and protein synthesis, as well as reduction of HbA1c (−1.16 ± 0.84%) and body weight gain (+0.27 ± 3.38 kg) [55]. Notably, beyond exerting these metabolic actions, insulin may participate in the pathophysiology of ED. Indeed, insulin resistance in diabetes-related ED appears to be closely associated with alterations in the sympathetic nervous system and NO synthesis, two essential systems involved in sustaining penile stiffness and tumescence [56,57]. However, when T1D rats underwent insulin treatment, in spite of increased body weight, erectile function was restored to the same extent as that of control littermates, together with enhanced expression of key markers of endothelial function within the cavernosal tissue (i.e., PECAM-1, phospho-/total eNOS, phospho-/total MYPT1) [58]. Evidence supporting insulin-associated amelioration of ED has previously been demonstrated in vivo. In particular, subcutaneous administration of insulin in T1D rats resolved the puzzle of higher intracavernosal pressure together with the restoration of sex hormone receptor expression levels [59]. Further, Yamanaka et al. described that the relief of diabetes-associated ED by insulin therapy also occurred via an anti-apoptotic mechanism as observed within the crura compartment [60]. By contrast, in another study, T1D rats exposed to multiple insulin injections showed an aggravation of erectile parameters as compared with control rats, principally due to a nitrergic degeneration (↓ NOS) and a decrease in testosterone levels, which were not reversed by insulin [61]. Diabetic rats treated with a single dose of insulin, however, had significant differences in the apoptotic index, smooth muscle components, and protein expression ratios of phospho-/total MYPT1, eNOS and Akt as compared with untreated rats (Figure 2) [61]. Moreover, a parallel arm, open-label, randomized, controlled study conducted in T2D men with ED demonstrated that the use of continuous subcutaneous insulin infusion, as compared to multiple daily injection therapy, for six months led to increased erectile function, with a reduction in ED severity or ED resolution associated with better glycaemic control [61].

Nevertheless, the complete recovery of erectile function by insulin treatment appears to be obtained by combining insulin administration with other therapeutic approaches. For instance, Zhou et al. demonstrated that ED improved after insulin treatment only exclusively via a cell-regeneration process driven by adipose stem cells (ASCs) [62]. In particular, ADSC injection alone per se led to a partial retrieval of penis activity (i.e., ↓ cell apoptosis, restored cavernous endothelium, etc.), but the favourable changes in penile tissue with functional recovery of erection were achieved only after insulin administration, which led to a reduction in AGE products within the penis compartment as observed in rats with T1D [62]. Similarly, Wang et al. provided evidence that insulin monotherapy partially restored erectile function and histological changes in rats with T1D, but better control of sexual parameters was obtained by associating glycaemic control with anti-oxidant therapy [63].

Overall, these results provide remarkable evidence that improvements in glycaemic control with insulin therapy could assist in the recovery from diabetes-associated ED. Recent findings, however, suggest that single monotherapy does not sustain the full restoration of penile activity to a near-normal status. A synergistic action of insulin and inoculations of ASCs could be a hopeful approach to warrant a complete remission of ED. 

### 3.2. Sulfonylureas

For several years, sulfonylureas (SUs) have been the most commonly prescribed medications for the treatment of T2D, being able to ameliorate glycaemic control reducing HbA1c levels (−1.0 ± 1.1%), despite induction of weight gain (+ 1.2 ± 2.3 kg) [64,65]. SUs are known to act as insulin secretagogues through the activation of SU receptor 1 on the cell surface of pancreatic β-cells. Following SU receptor 1 activation by these compounds, the inhibition of adenosine triphosphate (ATP)-sensitive K+ channels (KATPs) occurs, determining cell membrane depolarisation and resulting in exocytosis of insulin-containing granules [66]. Notably, the expression of KATPs was also found in different extra-pancreatic tissues, including the corporal smooth muscles, which physiologically regulate muscle tone and relaxation of penile resistance arteries to promote erection [67]. Even though the presence of these SU-targeted ion channels within the penile compartment is known, data on the effects of SUs in ED are currently inconsistent.

Insuk et al. demonstrated the presence of a subtype of KATPs within human corporal smooth muscle, i.e., Kir6.1–Kir6.2, whose location was found near to the SUR2B subunit [68]. In the same study, K+ flux within the human corpora cavernosum after stimulation with selective KATP activators was completely counteracted in the presence of non-selective SUs (i.e., glibenclamide and glimepiride), resulting in an inhibition of muscle vasodilatation [68,69,70]. 

Similarly, Rubio et al. observed that SUs (e.g., glibenclamide) block the myogenic tone and prevent muscle relaxation evoked by KATP openers; however, this effect was lost when SUs were administered together with PDE5i (i.e., sildenafil), indicating that KATP channels could not be involved in the relaxations induced by cGMP-elevating agents [71,72]. By contrast, in a cross-sectional controlled clinical study, glibenclamide-based therapy significantly triggered an increase in total and free testosterone levels, SHBG levels, sex drive, and erectile function in individuals with T2D [73]. In this setting, SUs appeared to sustain sex drive by direct stimulation of testosterone synthesis in men with T2D and hypogonadism [74,75]. 

Noteworthy, several retrospective studies have revealed that men with severe hypoglycaemia use illegal sexual enhancement products together with undeclared medication errors based on SU therapy (i.e., glibenclamide, glyburide), thus highlighting a possible positive effect of these insulinotropic drugs on sexual function [76,77]. Whether the potential therapeutic effects of SUs against ED development could be explained by the prevention of endothelial and vascular functionality is still poorly understood. In a recent randomised control trial, glibenclamide did not alter endothelial function when administered to men with T2D without cardiovascular complications as well as hypertensive diabetics, in whom its administration was found to worsen blood pressure [78,79]. Nevertheless, a novel SUs compound (I4) displayed greater alleviating effects on cardiovascular complications than glimepiride in T2D rats and significantly improved in vitro NO synthesis and NOS activity with final amelioration of endothelial dysfunction markers (Figure 2) [80]. 

In concert, these data highlight that further studies are needed to elucidate the controversial effects of SUs on endothelial cell functions and, consequently, on ED. Differences in SU selectivity could be the rationale behind the different outcomes observed.

### 3.3. Metformin

Metformin still represents one of the first-line medications for the management of T2D [81]. In addition to its well-known glucose-lowering effect (HbA1c reduction of −1.26 ± 0.02%), metformin favours some body weight loss (−2.5 kg) with a reduction in visceral fat area in association with multiple endocrine variations [2,82,83,84]. Interestingly, several in vitro studies have also ascribed anti-inflammatory and anti-atherogenic properties to this compound in terms of the prevention of oxidative stress and the amelioration of vascular and endothelial functions under stressor stimuli as ROS and inflammatory cytokines [85,86]. Particularly, novel insights have revealed that metformin restores the expression of two essential mediators of vasodilatory responses (nNOS and eNOS) within penile tissue of obese rats, thus suggesting potential implications in the treatment of ED [87]. Additionally, under metformin-based therapy, obese mice experienced a restored corpus cavernosum erectile response, together with attenuated norepinephrine-dependent sympathetic activities and decreased intracavernosal pressure [88]. 

As previously discussed, hyperglycaemia is a strong pathogenetic factor of penile dysfunction in individuals with T2D [1,89,90]. The extreme consequence of long-term exposure to high glucose levels is the accumulation of products of nonenzymatic glycation and angiotensin II, which accelerate the endothelial injury through oxidative stress and autophagy. In this scenario, metformin therapy appears to have a favourable effect against angiotensin II–induced ED in terms of the restoration of normal intracavernosal muscle tone and increased muscle relaxation according to an increase in eNOS activity as observed in a hypertensive rat model [91].

Angiotensin II was found to reduce the proliferation of CCSMCs, affecting the thickening of the penile vessel wall and damaging vasomotor function [92,93,94]. In a rat model, metformin therapy combined with icariside II, a PDE5i, restored the proliferation of CCSMCs and ameliorated NOS activity, leading to improvements in penile erectile function (Figure 2) [94]. The mechanism underlying the effects of metformin on ED is still under debate. Nevertheless, in vivo and in vitro evidence suggest the involvement of the AMP–AMP deaminase (AMPD) axis. In particular, metformin is known to favour AMP breakdown by inhibiting AMPD [95,96], a cytosolic enzyme regulating the signalling of adenosine, a key neuronal mediator of penile vasculature function and a potent endogenous vasodilator [97]. Additionally, metformin reverted metabolic syndrome in mice being fed a high-fat diet in association with an amelioration of erectile function, probably through the regulation of adenosine signalling [98]. In particular, in both ex vivo and in vivo experimental conditions, metformin mostly inhibited adenosine degradation, thus improving its signalling in the corpora cavernosa [98]. Despite these interesting results, the ability of metformin to treat ED in humans is still unclear. Notwithstanding, a prospective, randomised, double-blind, placebo-controlled pilot study observed that non-diabetic men with ED, insulin resistance and a prior history of poor response to sildenafil had improved erectile function after metformin therapy in combination with sildenafil [99]. These data highlighted that insulin resistance might be a mechanism involved in ED, and its treatment might optimize the response to sildenafil, thus suggesting that diagnosis and treatment of insulin resistance might represent an initial management plan for ED [99]. 

Metformin intervention also appears to prevent testicular spermatogenic dysfunction observed under hypercaloric conditions. Indeed, mice fed a high-fat diet exhibited an amelioration of semen quality and restored endogenous hormone levels (i.e., testosterone) after metformin treatment, supporting the hypothesis that this compound could also preserve reproductive functions in the setting of ED [100]. Finally, data from patients with metabolic syndrome have demonstrated an association between metformin administration and increased androgen levels associated with an improvement in semen characteristics [101]. Nevertheless, further investigations are necessary to identify the molecular mechanisms by which metformin could act on the reproductive system, thus counteracting ED development.

### 3.4. Acarbose (ACA)/α-Glucosidase Inhibitors

ACA acts as a reversible inhibitor of pancreatic α-amylase and hydrolase, which are involved in carbohydrate catabolism, thus delaying glucose absorption in the gut and thus determining a weight-loss (−3.3 ± 3.7 kg) [102,103]. The growing number of side effects involving the gastrointestinal tract (e.g., diarrhoea, abdominal pain), however, has made it difficult to use these molecules in the clinical practice of T2D. The anti-hyperglycaemic action of ACA is slightly lower to that of metformin and SUs in terms of the reduction in HbA1c levels (−1.86 ± 1.10%) [103,104]. In addition, this drug could also reduce the incidence of newly diagnosed diabetes among individuals with impaired glucose tolerance [105]. Nevertheless, the cardiovascular effects of ACA are still being debated. In an international, multicentre double-blind, placebo-controlled randomised trial, ACA-based therapy demonstrated a cardioprotective role [106], possibly caused by the prevention of endothelial cell dysfunction [107] in both individuals with impaired glucose tolerance and new-onset T2D.

Conflicting data on the efficacy of ACA against diabetic-related ED exist, even though this drug has shown anti-oxidant and anti-inflammatory properties [108,109,110]. An in vivo study revealed that ACA attenuates vascular barrier dysfunction and improves the hyperglycaemia-induced inflammasome, thus highlighting its potential role in the remission of vascular pathology under diabetic conditions [111]. These results suggest that a pharmacological intervention based on ACA treatment could exert beneficial effects on ED. Recent in vivo findings in T1D rats have revealed that the benefits of ACA on ED were potentiated were animals fed with moringa seed and a leaf-fortified diet as reported by expression analysis of some ED-related biomarkers in the penile tissue (i.e., acetylcholinesterase (AchE), monoamine oxidases (MAO), adenosine deaminase (ADA), angiotensin I converting enzyme (ACE), thiobarbituric acid reactive substances (TBARS)) [109].

Taken together, these findings support the concept that a combination therapy based on lifestyle modifications plus ACA treatment could help to restore a normal sexual function, probably by reducing inflammatory responses and enhancing antioxidant pathways within the penile tissue. However, investigations in this regard are still too scarce to suggest use of α-glucosidase inhibitors in ED.

### 3.5. Thiazolidinediones

In the last decade, the pharmacological management of T2D has been improved by the discovery of new insulin sensitiser molecules such as thiazolidinediones (TZDs), also called ‘glitazones’, which able to ameliorate insulin sensitivity (HbA1c reduction of −2.0 ± 0.4%) [112] and pancreatic β-cells function by stimulating the activity of PPARγ, a critical target of the regulation of energy homeostasis. Among these compounds, rosiglitazone and pioglitazone are currently approved by the United States Food and Drug Administration as monotherapy or for use in combination with metformin or SUs, even though they are not appropriate for the correction of body weight (−0.03 ± 1.52 kg) [113]. In addition to their metabolic benefits, TZDs have also shown pleiotropic effects, particularly in regard to cardio-protection [114,115,116]. Indeed, the expression of PPARγ was also identified in vascular SMCs, where its activation by agonists reduced the release of ROS and pro-inflammatory molecules and prevented the occurrence of vascular damage typically observed in a hypertensive state [117]. In this scenario, pioglitazone modulated the vasculature contractility in vivo by interfering with endothelin-1–induced vasoactive effects, thus warranting the protection of vessel structure and function [118]. Whether or not the same pathways are involved in the TZD-induced amelioration of ED is still unclear. 

Notwithstanding, in vivo results reported that pioglitazone could enhance the response to oxidative stress as observed in the setting of corporal veno-occlusive dysfunction. In particular, low-dose pioglitazone attenuated fibrotic processes and oxidative stress within the corpora cavernosum of rats with T2D regardless of glycaemic control [119]. Then, the same authors highlighted that the amelioration of corporal compliance by pioglitazone in an ageing-related corporal veno-occlusive dysfunction model was due to the increase in penile relaxation through the inactivation of the Rho-kinase system, an important inhibitor of SMC relaxation and penile erection [120]. However, this molecule was unable to affect the ratio of collagen to SMCs within penile tissue; therefore, the remission of ED occurred by counteracting anti-erectile agents, but not fibrotic factors [120].

Additionally, animals with similar cavernosal nerve trauma experienced dose-dependent improvements in intracavernosal pressure after pioglitazone-based therapy together with increased expression levels of eNOS, nNOS and cGMP, culminating in the recovery of normal erectile performance (Figure 1) [121]. These findings were also confirmed by in vitro results, showing that pioglitazone resulted in the inhibition of Ca^+2^ influx into penile SMCs by blocking L-type voltage-dependent calcium channels [122] and upregulating eNOS within vascular endothelial cells, thus triggering vasorelaxation [123].

Pioglitazone appears to preserve erectile activity when the corporal cavernosum undergoes neuronal failure. This was demonstrated in rats with bilateral cavernosal nerve crush injury [124]. In particular, when pioglitazone was administered both pre- and post-surgically, both survival and regeneration responses within the neuronal pelvic compartment were activated with an increase in nitrergic penis-projecting neurons and upregulation of key neuroprotective molecules (e.g., neurotrophin, neuturin) [124]. Currently, the mechanism underlying these effects are still poorly investigated, even though some studies have established that the prevention of erectile failure by pioglitazone administration could occur via both anti-inflammatory and insulin-like growth factor I (IGF-I)-mediated pathways. Indeed, when IGF-I receptors (IGF-IRs) were inhibited by a selective antagonist in vivo, pioglitazone failed to show neuroprotective activity for peripheral nerve regeneration within penile tissues in a rat model with bilateral cavernous nerve injury [125]. However, when pioglitazone was infused alone, an amelioration of nNOS protein expression was observed together with an upregulation of key downstream factors of IGF-IR signalling that are typically involved in the regulation of cell proliferation (Figure 2) [125]. Therefore, these novel results open a new window on a potential direct action of TZDs in the resolution of ED; however, it remains to be clarified whether these effects could improve erectile function under diabetic conditions.

Although the androgenic actions of pioglitazone were poorly analysed, a double-blinded study contended that treatment with pioglitazone in men with moderate-to-severe ED and lower sildenafil responsiveness resulted in the improvement of erectile function scores and increased sildenafil response, without changes in testosterone, glucose levels and other clinical parameters [126]. 

Conversely, in vivo studies reported that sildenafil abolished the vasorelaxant effects of both pioglitazone and rosiglitazone, interfering with potential beneficial effects of these antidiabetic drugs on ED and probably exerting an aberrant activity on certain ATP-dependent ion transporters through the enhancement of glucose-/glycolysis-dependent contraction-facilitating processes [127].

In concert, these results provide evidence that TZDs, particularly pioglitazone, could act as a regenerative agent to facilitate functional recovery of erectile activity and as a anti-vasoconstrictor molecule in the presence of severe cavernosal impairment. Nevertheless, further studies should elucidate the interactions of TZDs with drugs commonly used in sexual dysfunction that could compromise treatment outcomes and compliance.

### 3.6. GLP-1RAs and DPP-4i

Incretin-based therapies, including DPP4is, also called gliptins, and incretin mimetics, such as GLP-1RAs, are widely used in the clinical treatment of T2D due to their ability to improve hyperglycaemia (HbA1c reduction of −2.3 ± 1.2%) [128], in association with improved survival and function of β-cells and suppression of glucagon secretion [129,130]. Both GLP-1RAs and DPP4is showed cardioprotective actions in animal models of myocardial ischemia and ventricular dysfunction through not fully known molecular mechanisms, although the results of cardiovascular outcome trials in human subjects with T2D and increased cardiovascular risk have demonstrated a cardiovascular benefit only for GLP-1RAs [131,132,133]. Over time, these latter agents have also shown various biological effects beyond controlling glycaemic excursions in extra-pancreatic tissues [134], including weight-lowering efficacy (−2.2 ± 2.8 kg) [128,135,136,137], which is achieved through the regulation of satiety responses via central mechanisms [138], and, above all, cardioprotective and vasodilatory effects [139,140]. Indeed, endothelial cells, including human coronary artery endothelial cells and human umbilical vein endothelial cells, express the GLP-1 receptor (GLP-1R) [141,142]. In addition, several in vivo and in vitro studies have suggested that GLP-1 and its analogues directly control endothelial function [143,144] by stimulating the AMPK–eNOS axis and NO production [145,146]. Recently, Cai et al. found that GLP-1 is able to prevent oxidative stress-induced dysfunction and autophagy in human endothelial cells, and that the protective effects of GLP-1 might be dependent upon downstream restoration of the epigenetic factor histone deacetylase 6, a downstream molecular effector of EKR1/2 induced by oxidant injury, suggesting the potential therapeutic application of GLP-1 in the prevention and treatment of endothelial damage induced by oxidative stress in subjects with T2D [147]. Treatment with liraglutide, a widely used GLP-1RA, for four weeks significantly decreased body weight, blood cholesterol and triglycerides and improved the concentration-dependent acetylcholine-mediated response of the distal thoracic aorta in a *Ldlr^−/−^* mouse model of atherosclerosis. This liraglutide-mediated improvement in endothelial function might be due to changes in the expression levels of genes related to inflammatory pathways, extracellular matrix modulation and vascular smooth muscle cell differentiation [148]. Moreover, liraglutide was able to improve vascular dysfunction also by regulating a cAMP-independent PKA–AMPK pathway in perivascular adipose tissue and by the increasing anti-oxidant capability, as demonstrated in high-fat diet-induced obese mice [149].

Importantly, endothelial dysfunction represents a key event not only in the development of atherosclerosis, but also of ED since impaired NO production by the endothelium and/or increased inactivation of NO by ROS is fundamental in the induction of an erection [150]. In this regard, Yuan et al. recently demonstrated that liraglutide could improve erection function in diabetes-induced ED by regulating smooth muscle dysfunction, oxidative stress and autophagy, independently of a glucose-lowering effect, in a rat model of T1D and in CCSMCs [151]. In addition, GLP-1R inhibition abolished the beneficial functions of liraglutide treatment [151].

Since the GLP-1R is also expressed in the testes, the direct impact of GLP-1RAs on the reproductive system could also be derived from activation of the testicular GLP-1R. Indeed, when GLP-1R was genetically ablated in vivo, mice exhibited impaired glucose tolerance in association with significant decreases in adrenal, testis and seminal vesicle weights [152]. The impact of GLP-1 agonism on testes was also assessed in a high-fat diet-induced obesity mouse model characterised by reduced serum levels of testosterone, impairment of sperm quality and increased inflammation of the testes. Meanwhile, exenatide treatment reduced body weight, as well as the expression of pro-inflammatory cytokines, and improved the quality of sperm to the level of controls [153]. 

Considering human studies, a recent retrospective study has evidenced that liraglutide, in addition to lifestyle changes and metformin and testosterone therapy, allowed not only reaching glycemic target and lowering body weight, but also obtaining a considerable improvement of ED in diabetic obese men with overt hypogonadism [154]. Similarly, short-term combined treatment using exenatide and metformin was found to be superior to glimepiride and metformin in the correction of sexual dysfunction in patients with obesity and T2D [155]. Nevertheless, current data on GLP-1 and its analogues and reproduction are still scarce and controversial. In a case report, the use of liraglutide was found to cause interrupted sperm production in a 35-years-old man experiencing primary and idiopathic infertility, which was completely restored after five months of liraglutide withdrawal [156]. Therefore, further experimental studies are needed to clarify the effects of incretin mimetics on the male reproductive system.

It should be noted that the exact molecular processes by which GLP-1 may restore erectile performance are however still unknown. New insights have highlighted the importance of the Ras homolog gene family (RhoA)–Rho-associated protein kinase (ROCK) pathway in maintaining a flaccid penile state; notably, the inhibition of RhoA–ROCK signalling is able to improve ED, even when associated with hard-to-treat causes, such as diabetes, suggesting that RhoA–ROCK could represent a new therapeutic target [157]. Indeed, as previously discussed, liraglutide exerted protective effects on ED associated with the regulation of smooth muscle dysfunction, ROS production, and autophagy by regulating the RhoA–ROCK pathway (Figure 2) [151]. Another potential molecular mechanism by which GLP-1RAs might improve ED involves the combined downregulation of NADPH oxidase and increased NO production via eNOS and nNOS: in an animal model of methylglyoxal-induced corpus cavernosum dysfunction, chronic treatment with exendin-4 exerted a protective effect on this pathway independently of its glucose-lowering effect [158].

DPP4is are another promising class of glucose-lowering medications widely used in T2D therapy (HbA1c reduction of −0.54 ± 1.22%) [159]. These agents extend the half-life of endogenous incretin hormones by preventing their proteolytic cleaving, resulting in metabolic improvement [160]. Besides the insulinotropic ability of DPP4is, these drugs could interfere with the degradation of key players that regulate the trafficking of circulating endothelial progenitor cells released from bone marrow [161], including SDF-1α [162], substance P [163,164], and pituitary adenylate cyclase–activating polypeptide (PACAP), a peptide isolated in the hypothalamus that enhances gonadotropin release and improves sex steroid levels. PACAP also has vasorelaxant effects, and, similarly to substance P, improves vascular endothelial growth factor levels [165,166]. Interestingly, vildagliptin, a DPP4i, was recently shown to inhibit the development of endothelial dysfunction and to prevent atherogenesis in non-diabetic apolipoprotein E–deficient mice [167]. In addition, Ma et al. reported that saxagliptin, another DPP4i, suppressed oxidised low-density lipoprotein cholesterol (ox-LDL)-induced endothelial dysfunction in human vascular endothelial cells, by inactivating JNK, AP-1 and NF-κB signalling [168]. Therefore, DPP4is could be used in vascular pharmacotherapy, potentially resulting in amelioration of ED.

However, similarly to GLP-1RA, a case report described a deterioration of semen quality following three months of sitagliptin therapy in a subject with T2D, with semen quality recovering upon discontinuation of the drug [169]. However, similarly to GLP-1RA, a case report described a deterioration of semen quality following three months of sitagliptin therapy in an individual with T2D, with semen quality recovering upon discontinuation of the drug [169].

Taken together, these data highlight that incretin-based therapies have the potential to be included in the pharmacotherapy of ED regardless of their glucose regulatory effects. Despite the paucity of mechanistic studies in humans, incretin mimetics could evoke antioxidant responses via the RhoA–ROCK pathway, thus improving the homeostasis of CCSMCs and erectile activity. On the other hand, DPP4is could enhance the signalling pathways regulating endothelial function and vascular repair via SDF-1α, substance P, and PACAP, thus participating in the improvement of erectile performance.

### 3.7. SGLT2is

SGLT2is, also called gliflozins, represent another class of anti-hyperglycaemic agents currently used in T2D treatment; these medications are able to reduce hyperglycaemia (Hb1Ac reduction of −0.8 ± 1.4%) through the inhibition of glucose reabsorption in the proximal convoluted tubule of the kidney and the increase of glycosuria [170,171,172]. 

Large randomised trials of SGLT2is have shown reductions in cardiovascular events (particularly rates of hospitalisation for heart failure) in patients with T2D and in those with heart failure with reduced ejection fraction with or without diabetes [173]. Positive effects on body weight (−2.8 ± 4.9 kg) and the cardiovascular system manifest rapidly and, for this reason, are unlikely to be related only to the improvement in glycaemic control [172]. Indeed, SGLT-2i have also shown positive effects on multiple cardiovascular disease risk factors, including high blood pressure and obesity, as well as on serum uric acid and albuminuria levels [174,175]. 

To date, numerous in vitro and in vivo studies have investigated the molecular mechanisms through which SGLT2is exert positive cardiovascular effects. In this regard, it has been recently demonstrated that empagliflozin, a selective SGLT2i, not only reduced glucose levels, but also attenuated endothelial dysfunction and atherogenesis and improved cardiac remodelling in diabetic apolipoprotein E–deficient mice and in an experimental model of metabolic syndrome, the obese ZSF1 rat [176,177]. In addition, mice with T2D treated with dapagliflozin, another widely prescribed SGLT2i, for eight weeks displayed significantly less arterial stiffness, improvements in endothelial and vascular smooth muscle dysfunctions and reductions in circulating markers of inflammation as compared with nontreated diabetic mice [178]. 

Data obtained from experimental models have been confirmed in human studies, as evidenced by a recent pilot study in which acute treatment of subjects with T2D with dapagliflozin significantly improved systemic endothelial function and reduced both renal resistive index and aortic stiffness [179]. These multiple nonglycemic effects reinforce the concept that SGLT-2i should be the preferred glucose-lowering drug in the management of subjects with T2D with heart failure [180,181,182]. Moreover, some researchers have explored the possible direct effects of SGLT2is on human endothelial cells. In particular, Uthman et al. showed that empagliflozin and dapagliflozin were able to restore NO bioavailability, by reducing ROS generation, in tumour necrosis factor–α-stimulated human coronary artery endothelial cells and human umbilical vein endothelial cells, without affecting eNOS expression and signalling, barrier function, or the expression of adhesion molecules such as VCAM-1 and ICAM-1 [183]. Data obtained from experimental models have been confirmed in human studies, as evidenced by a recent pilot study in which acute treatment of individuals with T2D with dapagliflozin significantly improved systemic endothelial function and reduced both renal resistive index and aortic stiffness [179]. These multiple nonglycemic effects reinforce the concept that SGLT-2i should be the preferred glucose-lowering drug in the management of individuals with T2D with heart failure [180,181,182]. Moreover, some researchers have explored the possible direct effects of SGLT2is on human endothelial cells. In particular, Uthman et al. showed that empagliflozin and dapagliflozin were able to restore NO bioavailability, by reducing ROS generation, in tumour necrosis factor–α-stimulated human coronary artery endothelial cells and human umbilical vein endothelial cells, without affecting eNOS expression and signalling, barrier function, or the expression of adhesion molecules such as VCAM-1 and ICAM-1 [183].

Since endothelial dysfunction represents a key event in the development of ED, as detailed above, SGLT2is are likely to favourably affect ED as well. The effects of chronic treatment with empagliflozin on ED have been investigated in a T2D rat model, the Goto–Kakizaki rat, in the presence or absence of acute intravenous injection of sildenafil [184]. The erectile response was significantly improved in diabetic Goto–Kakizaki rats treated with empagliflozin, relative to placebo, and this was associated with an improvement in cavernosal nitrergic relaxation, suggesting a positive effect of empagliflozin on the nerve injury (Figure 2). However, further investigations are needed to understand whether the positive effects of empagliflozin on ED are due to better glycaemic control and/or to a reduction in diabetes-associated inflammatory state, and/or alternatively to a direct effect on penile endothelial cells. 

## 4. Conclusions

To date, several glucose-lowering medications are available to treat subjects with T2D. Importantly, several experimental and clinical data have highlighted the ability of glucose-lowering drugs to improve endothelial dysfunction and preserve endothelial cell viability. In addition, since endothelial dysfunction represents a key event in the development of atherosclerosis, as well as of ED, the potential role of these agents in ED and the related molecular mechanisms are under investigation. Several lines of experimental evidence suggest potential effects of specific glucose-lowering medications in the therapy of ED. Metformin, TZDs and GLP-1RAs could represent the best choice to favour a partial recovery of normal erectile function in individuals with T2D (Table 1). Nevertheless, a multidrug intervention may potentially be associated with an additive effect, even though this has not yet been demonstrated. Further in vitro and in vivo studies, as well as high-quality clinical trials, are still needed both to define the mechanisms of action of such drugs and to validate their use in the management of ED. Furthermore, another important aspect relates to the need to evaluate the pharmacological interactions between glucose-lowering agents and other drugs used in the treatment of sexual dysfunctions. The treatment—or, more importantly, the prevention of this complication in individuals with T2D—must become fundamental since it both represents an early predictor of cardiovascular complications and affects the quality of life of these patients.

## Figures and Tables

**Table 1:** In vivo effects of mostly used anti-hyperglycaemic agents on HbA1c, body weight and erectile function.

Drug	Effect on HbA1c	Effect on Body Weight	Effect on Erectile Function (Demonstrated or Potential)
Insulin	↓↓	↑↑	?
SUs	↓	↑	↑/↓
Metformin	↓	↓	*↑*
Acarbose	↓	↓	?
TZDs	↓	*↑*	*↑*
GLP1-RA	↓↓	↓↓	↑
DPP4i	↓	=	?
SGLT2i	↓↓	↓	↑

DPP4i, dipeptidyl peptidase-4 inhibitors; GLP-1RA, GLP-1 receptor agonists; SGLT2i, sodium-glucose co-transporter-2 inhibitor; SUs, sulfonylureas; TZDs, thiazolidinediones. ↑ moderate increase, ↑↑ high increase, ↓ moderate decrease, ↓↓ high decrease, = no significant change, ? not yet clear.
